# A 10-Year Risk of Cardiovascular Disease among Patients with Severe Mental Illness at Mbarara Regional Referral Hospital, Southwestern Uganda

**DOI:** 10.1155/2020/2508751

**Published:** 2020-07-23

**Authors:** David Collins Agaba, Richard Migisha, Henry Mark Lugobe, Godfrey Katamba, Scholastic Ashaba

**Affiliations:** ^1^Department of Physiology, Mbarara University of Science & Technology, Mbarara, Uganda; ^2^Department of Obstetrics and Gynaecology, Mbarara University of Science & Technology, Uganda; ^3^Department of Physiology, King Ceasor University, Kampala, Uganda; ^4^Department of Psychiatry, Mbarara University of Science & Technology, Mbarara, Uganda; ^5^Department of Psychiatry, Kampala International University, Uganda

## Abstract

Cardiovascular disease (CVD) is a leading cause of morbidity and mortality worldwide. Patients with severe mental illness (SMI) are at a higher risk for developing CVD and have a higher risk for harboring factors related to CVD. In addition to the effects of antipsychotic medications, unhealthy lifestyle factors, such as poor diet, inadequate physical activity, cigarette smoking, and sedentary behaviors, are known to be risk factors that may contribute to poor cardiovascular health in patients with SMI. Early identification of individuals at elevated risk of CVD is essential so that dietary and lifestyle modifications or pharmacological interventions can be prescribed to alleviate the risk of cardiovascular disease. The objective of the study was to determine the 10-year risk of cardiovascular disease among patients with severe mental illness at Mbarara Regional Referral Hospital, southwestern Uganda. We conducted a cross-sectional study at the outpatient mental health clinic of Mbarara Regional Referral Hospital, between October 2018 and March 2019. We used the Globorisk CVD risk score to estimate the 10-year risk of CVD among patients with SMI, using the online Globorisk calculator. Participants were then assigned to one of three categories depending on their 10-year CVD risk score: <3% (low), 3–10% (intermediate), and >10% (high). We calculated the risk scores of 125 participants aged 40-74 years. Most of the participants were female 75 (60%), had a diagnosis of bipolar disorder 75 (60%), and had mental illness for ≥10 years 57 (46%). Eighty five percent (85%) of the participants had intermediate to high 10-year risk of CVD (64% with intermediate and 21% with high risk). The average risk score was significantly higher in males compared to females, 8.82% versus 6.43%, *p* = 0.016. We detected a high 10-year risk of CVD in a significant proportion of patients with SMI in southwestern Uganda. We recommend lifestyle modifications and pharmacological interventions to reverse risk or delay progression to CVD in this patient population.

## 1. Introduction

Cardiovascular disease (CVD), which includes ischaemic heart disease and stroke, is a leading cause of morbidity and mortality worldwide [1]. CVD is more prevalent among patients with severe mental illness (SMI) compared to the general population resulting in reduced life expectancy and increased morbidity and mortality in this population [2–5]. As previously reported [6], patients with SMI have a 78% higher risk for developing CVD and a 53% higher risk for harboring factors related to CVD [6]. The increased risk of CVD among patients with SMI is attributed to unhealthy lifestyle factors such as poor diet, inadequate physical activity, cigarette smoking, alcohol consumption, and sedentary behaviors [7, 8]. Additionally, biological mechanisms, including autonomic nervous system (ANS) dysfunction, hypothalamic-pituitary-adrenal (HPA) axis dysregulation, inflammation, lipid pattern abnormalities, oxidative stress, increased platelet reactivity, and obesogenic effects of psychotropic medication, have been proposed [9–12].

Early identification of individuals at elevated risk of CVD is necessary so that lifestyle modification or pharmacological interventions can be initiated to alleviate the risk of disease [13]. Current recommendations on the prevention of CVD in clinical practice emphasize the need to base interventions on an assessment of the individual's total burden of risk rather than on the level of any particular risk factor [14, 15]. This is because most people who develop CVD have several risk factors which interact to produce their total risk. CVD prevention guidelines recommend the use of CVD risk scores to guide treatment decisions for primary prevention in people who do not yet have clinical manifestations of CVD [16, 17]. Several prediction risk equations for CVD are available including the Framingham risk score [18], ASCVD (Atherosclerotic Cardiovascular Disease) risk score [19], QRISK [20], and others but have not been validated for use in low resource settings. Because of this, CVD risk assessment is not routinely done among patients with SMI in low resource settings, yet these patients are at risk of premature morbidity and mortality from CVD. We therefore used a novel risk score, the Globorisk score [21], a score that has been validated for use in low resource settings including Uganda, to determine the 10-year risk of CVD among patients with SMI at Mbarara Regional Referral Hospital (MRRH), southwestern Uganda.

## 2. Materials and Methods

This study was part of a bigger cross-sectional survey that was conducted at the outpatient mental health clinic of MRRH between October 2018 and March 2019 to evaluate metabolic syndrome among patients with SMI [22]. We enrolled participants ≥ 18 years of age, who were in remission phase or who had recovered from an acute episode of mental illness. We included all patients who had a diagnosis of SMI (bipolar disorder, schizophrenia, or major depression) as confirmed by the attending psychiatrist. The study excluded pregnant women as pregnancy would affect the interpretation of waist circumference and could potentially confound blood pressure findings in case of pregnancy-induced hypertension.

Sociodemographic and clinical data were captured using a structured interviewer-administered questionnaire. The study variables of interest included age, weight, height, sex, education level, residence, marital status, history of smoking, history of alcohol use, psychiatric diagnosis, duration on psychotropic medication, fasting blood glucose, and fasting total cholesterol. The outcome variable was the Globorisk 10-year risk score.

Weight was measured using a calibrated seca weighing scale (seca 762, GmbH & Co. KG, Hamburg, Germany) to the nearest 0.5 kg with the participant not wearing shoes and heavy clothes whereas height was measured using a stadiometer to the nearest 0.5 cm with the participant standing upright with the heel, buttock, and upper back along the same vertical plane. The details of the methodology have been described elsewhere [22].

### 2.1. Laboratory Tests

The laboratory tests were fasting blood sugar and fasting total cholesterol. Fasting blood sugar in mmol/l was measured using a FreeStyle Optium Xceed glucometer (Abbott, Witney, Oxfordshire, UK) after at least 8 hours of fasting using capillary blood obtained by finger prick. Fasting total cholesterol was tested from three mls (3 mls) of venous blood drawn from the cubital fossa after at least 8 hours of fasting using an ELITechGroup PIT-CHSL-4-v21 (09/2016) machine following the enzymatic-colorimetric method. All procedures were done following standard operating procedures as previously described [22]. Those who had not fasted for 8 hours at the time of recruitment were only included in the study if they were willing to wait till they completed 8 hours of fasting.

Diabetes mellitus was defined as fasting capillary whole blood glucose concentration ≥ 7.0 mmol/l or currently on medication for diabetes mellitus [23, 24]. We then determined the Globorisk score of each participant using an online Globorisk calculator by filling in each participant's age, sex, total cholesterol, smoking status, and diabetes mellitus status. Participants were then assigned to one of three categories depending on their 10-year CVD risk score: <3% (low), 3–10% (intermediate), and >10% (high).

### 2.2. Data Handling and Analysis

The data were cleaned, then double entered into EpiData 3.1, after which they were exported to STATA version 12 (StataCorp, College Station, Texas, USA) for analysis. Descriptive analysis of all variables was done, describing continuous variables as mean ± SD and categorical variables as percentages. Categorical and continuous Globorisk predictors and categories were compared based on sex using the chi-square test and Student *t-*test, respectively.

### 2.3. Ethics

Ethical clearance for the study was obtained from the Mbarara University of Science and Technology Research Ethics Committee (No. 07/08-18) and the Uganda National Council for Science and Technology (HS 2548). Data were collected by research assistants trained to handle data with confidentiality. We respected the guidelines of Helsinki regarding research with humans [25]. Written informed consent was obtained by research assistants from all participants.

## 3. Results

A total of 304 participants were enrolled in the study. For this analysis, only 125 were considered since the Globorisk score is age specific (40-74 years).

Most of the participants were aged 40-60 years (88.8%), were female (60%), were married/living with a partner (52%), and lived in rural areas (82.40%).

Bipolar disorder was the commonest diagnosis (60%), and the majority of the participants had been with mental illness for 10 years or more (45.60%). The majority of the participants were on an antipsychotic (80.00%) with the biggest percentage on typical antipsychotics (91%) as shown in Table 1.

The Globorisk predictors and the 10-year risk of CVD of the participants are shown in Table 2. The average age was 50.61 ± 8.00 years, and there was no statistically significant difference in age between males and females. The majority of participants were nonsmokers (84%). The proportion of participants with diabetes mellitus was 13.60%. The majority of participants had an intermediate 10-year risk of CVD (64%) with an average risk score of 7.38 ± 6.10%, being higher in males than females. One in five participants (20.80%) was at a high 10-year risk of CVD.

## 4. Discussion

Our results indicate that patients with SMI at Mbarara Regional Referral Hospital are at an increased 10-year risk of CVD, with 80.84% having intermediate to high risk, the risk being higher in males compared to females. This could be attributed to the high prevalence of metabolic syndrome (MetS) of 23.51% previously found in the same population [22]. MetS has been found to play a key role in developing CVD [26]. A diagnosis of MetS is helpful in screening patients with SMI at a higher risk of CVD as it may lead to interventions to reduce disease burden and increase longevity; but because the criteria for MetS omits risk factors for CVD such as gender, age, and smoking status, MetS does not reflect a continuous spectrum effect on CVD risk [27]. This calls for multivariable risk assessment tools, such as the Globorisk score to estimate CVD risk.

The average CVD risk in this study was 7.38% which compares with the risk found in other studies elsewhere in China (6.7%) [28] and Taiwan (4.7%) [27]. The differences in the CVD risk could be attributable to the different risk prediction scores used in these other studies which mainly used the Framingham risk score (FRS) which uses a wider age range, 20-70 years, compared to the Globorisk which uses a narrower age range of 40-74 years. The FRS includes people below 40 years whose risk for CVD is generally lower and therefore could explain the low risk in these studies. The risk of CVD in our study was however lower than in the UK (10.4%) [29] and Toronto (8.9%) [30] probably because of the differences in socioeconomic status across these countries.

Our findings also found a higher 10-year risk of CVD in male participants (8.82%) compared to females (6.43%), *p* = 0.016, a finding found in other studies [28, 31] probably because male patients are more likely to live risky lifestyles of smoking and alcohol consumption. In addition, oestrogen whose levels are higher in females compared to males is believed to be cardioprotective [32] through modulation of vascular function, inflammatory response, cardiac myocyte and stem cell survival, and slowing the development of hypertrophy [33].

Our findings emphasize the need to screen all patients with severe mental illness regularly for CVD risk factors and to manage these risk factors aggressively, in addition to encouraging healthy lifestyles. Early identification of individuals at elevated risk is essential so that lifestyle modifications or pharmacological interventions can be prescribed to alleviate the risk of disease [13]. There is evidence to support treatment with statins for dyslipidemia, metformin for weight loss, and bupropion for smoking cessation [34]. Statin therapy is recommended as part of the management strategy for primary prevention of CVD for adults at a high risk of developing CVD, and treatment should be initiated in adults who have a ≥20% 10-year risk of developing CVD [35].

Our study had some limitations. First, the study was conducted in one hospital and findings may not be generalizable beyond the population of patients attending the mental health clinic at MRRH. Second, we did not have a control group to compare the cardiovascular risk with people in the general population which limits the generalizability of our study findings to the general population. Third, we did not collect information on the dosages of the medications used and hence, we were not able to determine if the dosages of the medications used could have predisposed our study participants to the cardiovascular risk reported in our study.

## 5. Conclusion

Our study revealed an elevated 10-year CVD risk in a significant proportion of patients with SMI, with males having a higher risk than females. We recommend routine screening for CVD, as well as CVD risk-lowering interventions such as encouraging healthy lifestyles among patients with SMI in Uganda. We also recommend a comparative study to determine the relative risk of cardiovascular disease among patients with severe mental illness compared to the general population.

## Figures and Tables

**Table 1:** Demographic and clinical characteristics of the participants (*N* = 125).

Characteristic	No. of participants (*n*)	Percentage (*n*/*N*) %
Age category		
40-60	111	88.80
60-74	14	11.20
Sex		
Male	50	40.00
Female	75	60.00
Weight, kg (mean ± SD)	72.26 ± 15.60	NA
Height, m (mean ± SD)	1.64 ± 0.08	NA
Marital status		
Single	15	12.00
Married/living with partner	65	52.00
Divorced/separated/died	45	36.00
Education level		
Never attended	20	16.00
≤secondary	77	61.60
Tertiary/university	28	22.40
Employment		
Unemployed	18	14.40
Employed	107	85.60
Area of residence		
Rural	103	82.40
Urban	22	17.60
Mental illness		
Bipolar disorder	75	60.00
Schizophrenia	33	26.40
Depression	17	13.60
Antipsychotic medication, yes	100	80.00
Antipsychotic class (*N* = 100)		
Typical	91	91.00
Atypical	09	09.00
Mood stabilizer (yes)	58	46.40
Antidepressant (yes)	30	24.00
Duration of mental illness (years)		
<5	45	36.00
5-10	23	18.40
>10	57	45.60
Duration of psychotropic medication (years)		
<5	52	41.60
5-10	48	38.40
>10	25	20.00

**Table 2:** Globorisk predictors and 10-year risk of CVD of the participants.

	Overall (*N* = 125)	Male (*N* = 50)	Female (*N* = 75)	*p* value
	*n*/*N* (%)	*n*/*N* (%)	*n*/*N* (%)	
Age, in years (mean ± SD)	50.61 ± 8.00	49.5 ± 8.42	51.35 ± 7.67	0.896
Smoking				0.135
No	105 (84.00)	39 (78.00)	66 (88.00)	
Yes	20 (16.00)	11 (22.00)	09 (12.00)	
Diabetes mellitus				0.523
No	108 (86.40)	42 (84.00)	66 (88.00)	
Yes	17 (13.60)	08 (16.00)	09 (12.00)	
Total cholesterol, mmol/l (mean ± SD)	203.74 ± 74.49	193.74 ± 70.22	210.42 ± 76.94	0.889
Average Globorisk score	07.38 ± 6.10	08.82 ± 6.97	06.43 ± 5.29	0.016
10-year Globorisk category				0.031
Low	19 (15.20)	05 (10.00)	14 (18.67)	
Intermediate	80 (64.00)	29 (58.00)	51 (68.00)	
High	26 (20.80)	16 (32.00)	10 (13.33)	

SD: standard deviation.

## Data Availability

The data used to support the findings of this study are available on request from the corresponding author.
